# ALDH1A3 serves as a predictor for castration resistance in prostate cancer patients

**DOI:** 10.1186/s12885-020-06899-x

**Published:** 2020-05-06

**Authors:** Shangqian Wang, Xiang Zhou, Chao Liang, Meiling Bao, Ye Tian, Jundong Zhu, Tongtong Zhang, Jie Yang, Zengjun Wang

**Affiliations:** 1 Department of Urology, The First Affiliated Hospital of Nanjing Medical University, Nanjing, 300 Guangzhou Road, Nanjing, 210029 China; 2 Department of Pathology, The First Affiliated Hospital of Nanjing Medical University, Nanjing, China; 3 Department of Urology, The Third Affiliated Hospital of Soochow University, The First People’s Hospital of Changzhou, Changzhou, China

**Keywords:** Castration resistance prostate cancer, Survival time, Aldehyde dehydrogenase, Androgen deprivation therapy, Resistance

## Abstract

**Background:**

Aldehyde dehydrogenase 1A3 (ALDH1A3) has been implicated in the survival and proliferation of prostate cancer cells.

**Methods:**

We retrospectively reviewed our patients with advanced disease on adjuvant hormonal therapy after prostatectomy. Time to castration resistance stage was documented. And Immunohistochemistry analysis for ALDH1A3 was performed for those patient samples on tissue microarray. Bioinformatics anslysis was used for RNA sequencing data of both primary prostate cancer and metastatic castration resistance prostate cancer (mCRPC) from online datasets. Crispr-Cas9 was used to knock out ALDH1A3 in prostate cancer luminal cells, and morphologic analysis as well as the Gene Set Enrichment Analysis (GSEA) were facilitated to discover the mechanisms of the resistance phenotype.

**Results:**

We found that the patients with ALDH1A3 low expression had shorter time to progression to castration resistance compared with those of higher expression group on adjuvant hormonal therapy after radical prostatectomy. The ALDH1A3 knockout cells gradually acquired resistance to androgen deprivation therapy, a few cells have been found in knockout group showing as that the spindle-like luminal cells in charcoal stripped medium. Furthermore, PI3K pathway activation has been confirmed by Western blot. The PI3K pathway inhibitor BEZ235 has been demonstrated that the acquired ADT resistance by ALDH1A3 down regulation could be rescued by PI3K pathway inhibitor.

**Conclusion:**

These results suggested a novel function for ALDH1A3 in development of mCRPC, and indicated PI3K pathway inhibitor has the potential in the treatment of a subgroup of mCRPC patients.

## Background

Androgen deprivation therapy (ADT) is the standard of care for advanced prostate cancer or progression after localized definitive treatment. However, most patients eventually progress to a condition known as castration-resistant prostate cancer (CRPC), characterized by lack of response to ADT. Despite the several treatment options for this stage of disease, the impact on overall survival is less than optimal and, most importantly, there is no reliable biomarker to predict the response of the treatment or resistance. As a result, no standard guidance is available to optimally sequence approved treatments for individual patients. Since 2005, the next-generation sequencing (NGS) technologies [1] have made it possible for us to better understand the molecular profiles of the cancer, which would provide evidence for clinical practice in oncology, such as diagnosis, prognosis, and treatment decisions. In prostate cancer, the Cancer Genome Atlas (TCGA) has revealed a molecular taxonomy of 333 primary prostate cancer [2]. In addition, the Stand Up to Cancer (SU2C) prostate cancer Dream Team also sequenced 150 metastatic castration resistant prostate cancer samples [3], which benefits a lot to investigate the mechanisms of castration resistance in this disease.

The aldehyde dehydrogenase family 1 member A3 (ALDH1A3) catalyzes the oxidation of retinal to the pleiotropic factor retinoic acid using nicotinamide adenine dinucleotide (NAD+). The level of ALDHs enzymatic activity has been regarded as a cancer stem cell (CSC) marker and seems to correlate with tumor aggressiveness [4] which has been investigated in pancreatic cancer [5], ovarian cancer [6], breast cancer [7], and high-grade glioma [8]. From our previous report [9], we found that ALDH1A3 highly expressed in the human prostate, specially in the luminal compartment. In the primary prostate cancer, the expression of this gene correlated with AR pathway and luminal markers. Furthermore, for those with advanced disease after radical prostatectomy who underwent adjuvant androgen deprivation therapy, negative ALDH1A3 expression predicted as shorter time for castration resistance upon hormonal therapy.

We found, surprisingly, that ALDH1A3 was down regulated in metastatic castration resistant prostate cancer from previous sequencing data [10]. Combing with our previous report, the low expression of ALDH1A3 might be related with regression of AR signaling pathway at castration condition. Strengthening with the proof reanalyzed from RNA sequencing of 150 mCRPC patients, ALDH1A3-low group seems to be related with lymph nodes metastases, and activation of PI3K pathway signaling. Furthermore, we confirmed this hypothesis with experiments, supporting that ALDH1A3 null could facilitate prostate cancer cells in castrated condition via PI3K pathway, but could be rescued by PI3K pathway inhibitor. These results provide evidence that ALDH1A3 could be a potential biomarker of castration resistant prostate cancer, supporting future clinical trial on overcoming the ADT resistance.

## Methods

### Patients and tissue microarrays

The protocol to generate the tissue microarrays (TMAs) in this cohort has been described in our previous report [9]. We retrospectively reviewed our patients with advanced disease on adjuvant hormonal therapy after prostatectomy. A total of 79 patients in our single center were included in this study. Those who has lost follow-up or benign tissue on the TMA were excluded. All these patients were performed laparoscopic radical prostatectomy with positive lymph nodes or positive margins followed by adjuvant hormonal therapy (LHRH analogs and bicalutamide as standard of care) between 2012 and 2014 at the urology department of the First Affiliated Hospital of Nanjing Medical University. All patients were recruited following informed consent, the protocol was approved by ethical committee of The First Affiliated Hospital of Nanjing Medical University. Progression to castration resistant prostate cancer (CRPC) defined as biochemical recurrence or metastasis on adjuvant hormonal therapy. For the staining score system, we have described the protocol in our previous report [9]. Briefly, For the staining score system [11], the percentage of positive tumor cells was determined by at least five areas at 400 magnification and assigned to one of the following five categories: 0 < 5%; 1:5–25%; 2: 25–50%; 3: 50–75%, and 4: > 75%. The intensity of immunostaining was scored as follows: 1 low, 2, moderate, and 3, strong. The IHC score for ALDH1A3 on prostate cancer slides was: low expression < 8, and high expression ≥8.

### Database and bioinformatics

Three datasets (Cornell [12], MSKCC [13], Michigan group [10]) on prostate cancer samples sequencing profiles were found and the RNA sequencing data in RPKM format were downloaded. The ALDH1A3 expression value for each samples in mCRPC group and primary cancer group were compared in each dataset. The results were shown by GraphPad software.

### Gene set enrichment analysis (GSEA) analysis

The RNA sequencing data were downloaded from SU2C database. The median value of RPKM for ALDH1A3 was used as cut-off value, any sample which is higher than the median value was determined as ALDH1A3^high^, the lower samples as ALDH1A3^low^. The GSEA analysis was performed according to the protocol which was previously described [14]. The Genesets were downloaded from the Molecular Signatures Database (MSigDB http://software.broadinstitute.org/gsea/msigdb/).

### Cell culture and Crispr-Cas9 knockout

The human prostate cancer cell line (LnCaP, VCaP) were purchased from the Cell Bank Type Culture Collection of the Chinese Academy of Sciences (Shanghai, China) and maintained in RPMI medium with 10% fetal bovine serum within a humidified atmosphere containing 5% CO2 at 37 °C.

We designed the guide RNA for ALDH1A3 from (http://crispr.mit.edu/), targeting the first exon. The sequence of the guide is as follows—ALDH1A3: 1- AGTTATGGCTACCACCAACG; 2-TAGTCTGCGGCGCACCGGCT; green fluorescent protein (GFP): GGCGAGGAGCTGTTCACCG. Then, we ligated the guide to the LentiCrispr-V2 system followed by Sanger sequencing validation [15]. Finally, we produced the lentivirus according to the protocol previously described [9]. After 2 days of the infection to the LnCaP and VCaP cells, the puromycin selection was performed. We performed Western Blot assay to validate the knockout efficiency after 14 days of infection. The cell numbers were counted based on the typan blue staining.

### Western blotting

The protein expression of ALDH1A3 by Western Blot assay was performed according to the protocol previously described. The antibodies against ALDH1A3 (Abcam, USA), Phospho-Akt (Ser473, Cell Signaling Technology, USA) and glyceraldehyde 3-phosphate dehydrogenase (GAPDH; Bioworld Technology, Inc., USA) were used in Western Blot assay in accordance with the manufacturer’s instructions.

### Statistical analysis

Differences in vitro experiment like cell numbers between groups were subjected to Student’s t test. *p* < 0.05 was considered to be statistically significant. All the statistical calculations were performed using GraphPad Prism v6.0 software (GraphPad Prism version 6.00 for Windows, GraphPad Software, La Jolla California USA, www.graphpad.com).

## Results

### ALDH1A3 negative predicted CRPC in patients on adjuvant ADT

**Fig. 1:**
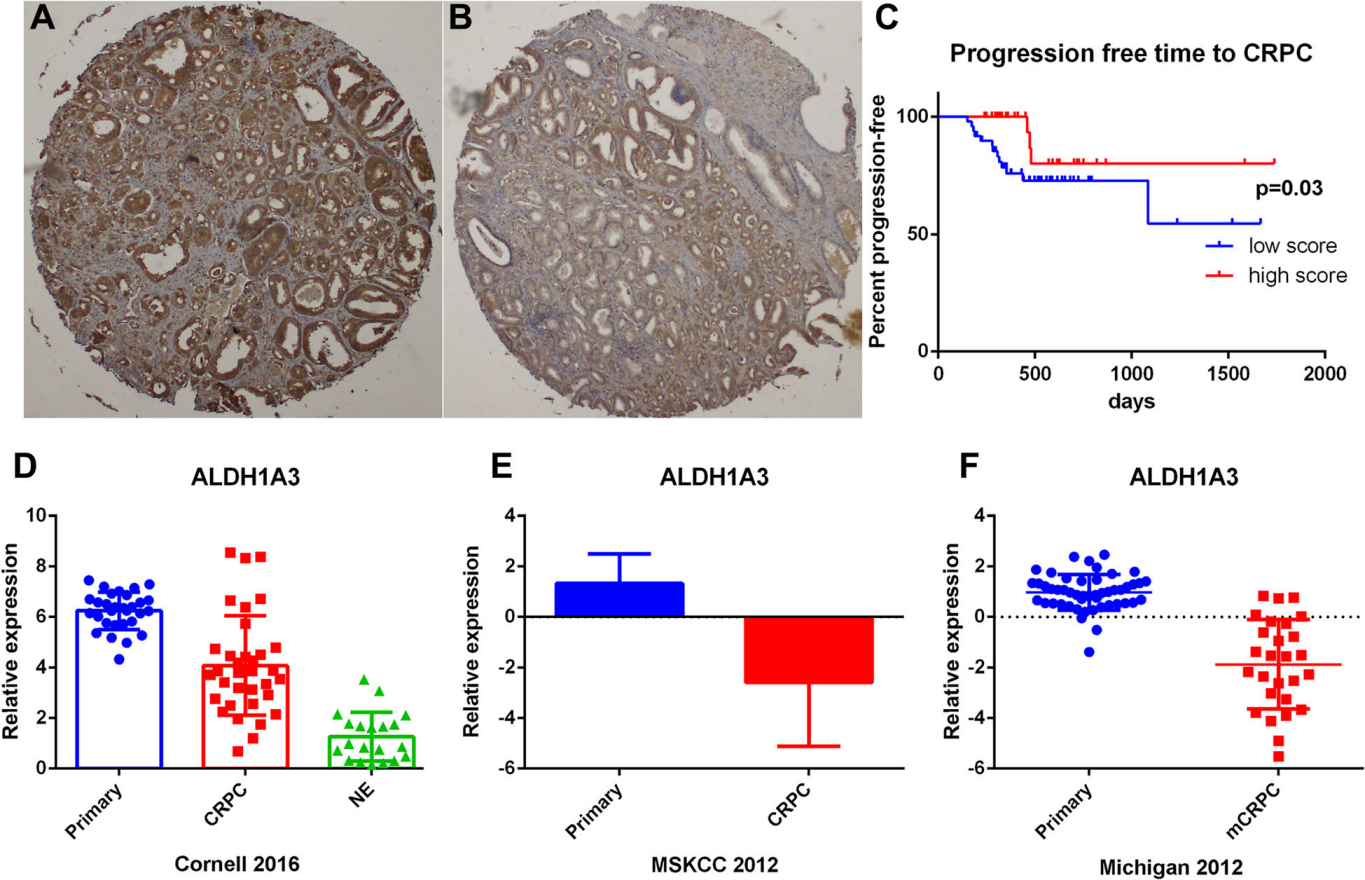
Differences of expression level for ALDH1A3 between primary and CRPC groups. **a**: IHC on TMA demonstrated high expression score of ALDH1A3. **b**: IHC on TMA demonstrated low expression score of ALDH1A3. **c**: Kaplan-Meier survival plot was used to indicate the time of progression to castration resistance between high vs low groups. **d**-**f**: The relative expression of ALDH1A3 is lower in CRPC compared with primary, but the neuroendocrine group ranking the lowest level. MSKCC group showed the same trend. Michigan data showed the same trend

Our earlier work has demonstrated that ALDH1A3 highly expressed in human prostate, which had a strong correlation with primary prostate cancer luminal signature and could be a potential biomarker of AR signaling pathway. Then we moved on to investigate its expression in the castration resistant prostate cancer. We retrospectively reviewed 79 patients with advanced disease who underwent radical prostatectomy followed by adjuvant hormonal therapy in our center. It was indicated that negative expression of ALDH1A3 predicted shorter time progression to castration resistance on adjuvant hormonal therapy (Fig. 1a-c). Besides, its expression is down regulated in three datasets. Beltran et al. performed a sequencing profiling on 114 metastatic samples from 51 CRPC and 30 neuroendocrine prostate cancer patients. The ALDH1A3 expression was lower in neuroendocrine and CRPC group compared with primary cancer (Fig. 1d). Another group performed RNA sequencing for primary prostate cancer and mCRPC samples, ALDH1A3 also down regulated in mCRPC samples (Fig. 1e). The data from Michigan group showed the same trend (Fig. 1f). All expression level of ALDH1A3 in these three datasets are defined as Z score or log2 value.

### ALDH1A3 signature has a strong correlation with prostate cancer progression, and PI3K signaling pathway

**Fig. 2:**
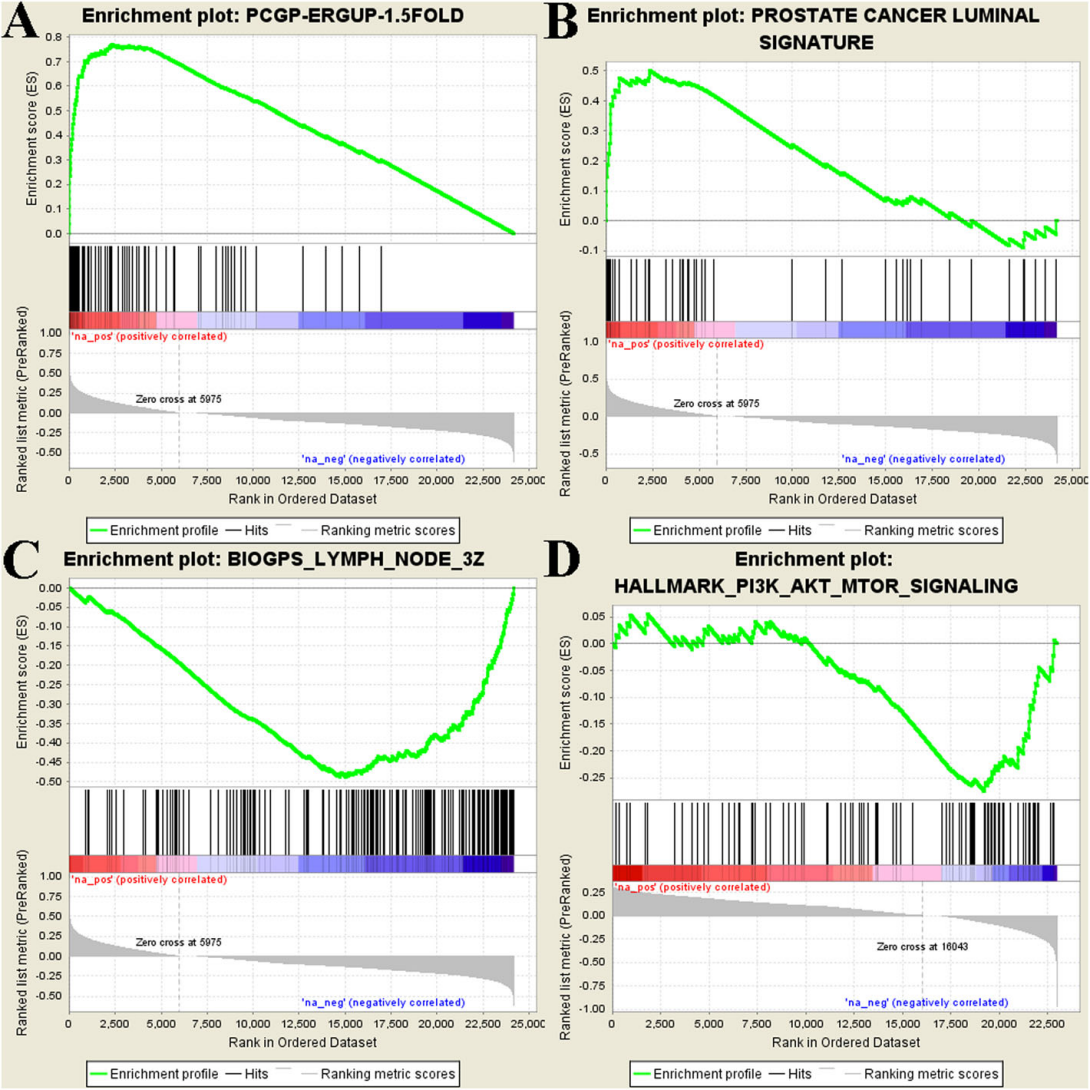
GSEA analysis for ALDH1A3 signature. **a**: ALDH1A3 has positive correlation with ERG up regulation. **b**: ALDH1A3 has positive correlation with prostate cancer luminal signature. **c**: ALDH1A3 has negative correlation with lymph node. **d**: ALDH1A3 has negative correlation with PI3K-AKT-mTOR signaling

A multi-institutional clinical sequencing infrastructure to conduct prospective transcriptome sequencing of bone or soft tissue tumor biopsies from a cohort of 150 mCRPC affected individuals was performed to establish a precision medicine framework for mCRPC. According to the expression level of ALDH1A3 in the dataset, we defined the cases above the median value of ALDH1A3 in the whole cases as ALDH1A3^high^, those whose expression level lower than the median as ALDH1A3^low^. We compared ALDH1A3^high^ and ALDH1A3^low^ cases to generate a differential expression gene list as ALDH1A3 signature (Table S1). The ALDH1A3 ranking the most significant changes in the whole list confirmed the results. The Gene Set Enrichment Analysis (GSEA) finally correlated ALDH1A3 signature with several biologic event. We found the ALDH1A3 signature had significant positive correlation with ERG signature and prostate cancer luminal signature, respectively (Enrichment score: 0.77, 0.5; Both *p* value: < 0.01) (Fig. 2a, b), and it had negative correlation with lymph nodes and PI3K-AKT-mTOR signaling pathway, meaning that ALDH1A3^low^ group might be associated with lymph nodes metastasis and PI3K-AKT-mTOR signaling activation (Fig. 2c, d).

### Down-regulation of ALDH1A3 causes ADT resistance in prostate cancer cells

**Fig. 3:**
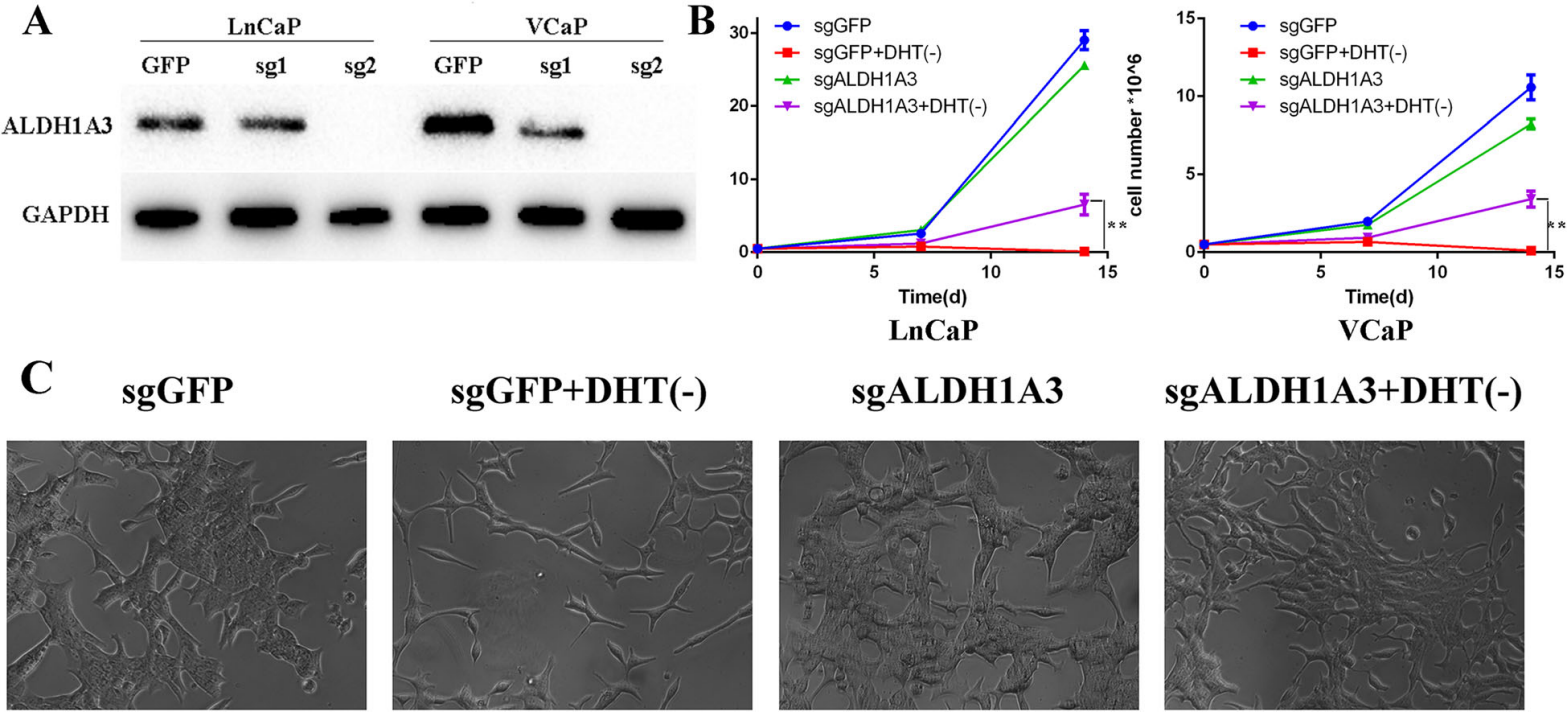
ALDH1A3 knockout facilitated luminal cells resistance to ADT therapy. **a**: Western blot validation for two candidate guides RNA of Cripsr-cas9, full-length blots are presented in Supplementary Figure 1–2. **b**: The cell numbers reflecting cell growth curve for different groups, sgGFP (control cells in normal medium), sgGFP+DHT(−) (control cells in charcoal stripped medium), sgALDH1A3 (Crispr-cas9 knockout ALDH1A3 cells in normal medium), sgALDH1A3 + DHT(−) (Crispr-cas9 knockout ALDH1A3 cells in charcoal stripped medium). Both in LnCaP and VCaP cells, there is significant difference between sgALDH1A3 and sgGFP in charcoal stripped medium at day 14 (*p* < 0.01). **c**: Morphologic observation for different groups

Based on the above results, we aimed to investigate the mechanisms of negative expression of ALDH1A3 in mCRPC samples. We designed small guide RNA targeting the functional exon of ALDH1A3 to knock out this gene on cell level to see the phenotype changes. After validation of the knock out efficiency (Fig. 3a, Fig. S1–2), we picked up the most potent sgRNA to target ALDH1A3 in LnCaP and VCaP cells, both of which are sensitive to androgen ablation treatment in vitro. We found that the growth rate of the control cells (targeting GFP) had no significant difference with ALDH1A3 knockout cells in normal medium. But in charcoal stripped medium which has already filtered androgen, the ALDH1A3 knockout cells, growing slowly, could be able to survive. As a result, the growth rate of ALDH1A3 knockout cells was significantly faster than the control cells in charcoal stripped medium (Fig. 3b). We also repeated the experiment in VCaP cells, and the results were consistent with those in LnCaP cells (Fig. 3b). We did the morphology observation for those LnCaP cells as well to predict the potential mechanisms of the ADT resistance. From Fig. 3c, At day 7, the control cells in medium without DHT showed spindle-like morphology and the cell number is low compared with those control cells in normal medium showing in aggregation or in cluster. However, it didn’t show any difference in ALDH1A3 knockout cells in DHT-free medium and in normal medium. At day 14, 95% of the control cells in DHT-free medium had been dead, whereas there were 30–40% of the ALDH1A3 knockout cells still alive.

### ALDH1A3 knockout facilitates castration resistance through PI3K-AKT-mTOR signaling pathway

**Fig. 4:**
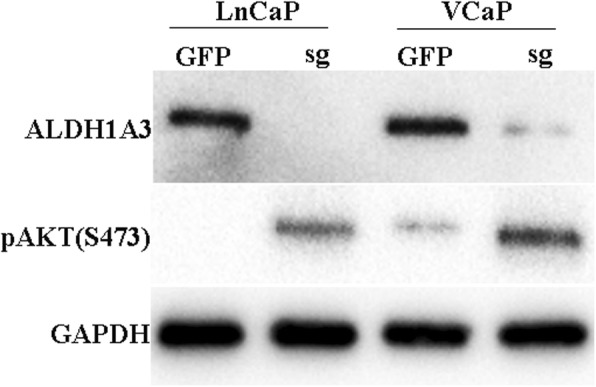
Western blot for sg-ALDH1A3 in LnCaP and VCaP cells, the pAKT was up regulated in sgALDH1A3 group. Phospho-AKT was up-regulated in ALDH1A3 knockout group, full-length blots are presented in Supplementary Figure 3, 4, 5

**Fig. 5:**
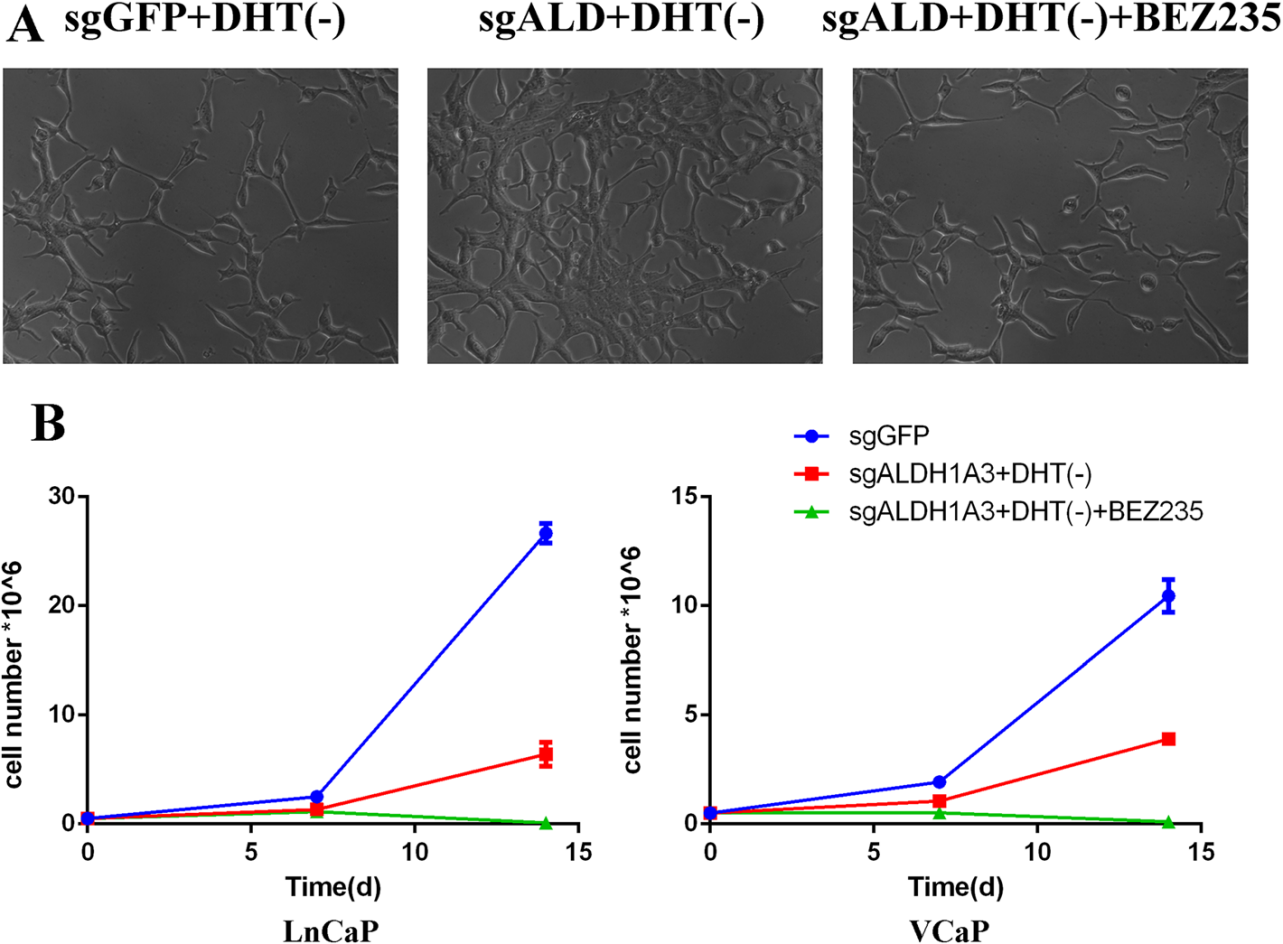
BEZ235 treatment rescued the resistance through PI3K pathway inhibition. **a**: Morphologic observation for BEZ235 treatment compared with the resistance phenotype. **b**: The growth curve documented the cell numbers of different groups, BEZ235 treatment rescued the ADT resistance in sg-ALDH1A3 group

Based on the above GSEA results, the ALDH1A3 signature negatively correlated with PI3K-AKT-mTOR signaling pathway. We speculate that the ALDH1A3 loss could activate the PI3K pathway. In order to validate this hypothesis, we did Western blot assay to test the PI3K pathway activation to compare the ALDH1A3 wild type cells and knockout cells. The blotting showed that both in LnCaP and VCaP cells, phospho-AKT had been elevated in ALDH1A3 knockout group (Fig. 4, Fig. S3, 4, 5). Next, in order to determine the relationship between castration resistance and PI3K signaling pathway activation by ALDH1A3 knockout, We performed rescue assay to block PI3K signaling pathway by using PI3K signaling inhibitor BEZ235. The results demonstrated that 500 nM BEZ235 treatment after 48 h could rescue the resistance by ALDH1A3 knockout. In terms of the morphology analysis, LnCaP cells in BEZ235 treatment and DHT-free group showed spindle-like morphology, similar with control cells in the DHT-free medium (Fig. 5a). Those cells showed less aggressive phenotype and finally detached from the bottom of the plate. The growth rate of ALDH1A3 knockout cells showed 30% higher than the wild type cells but was inhibited totally by BEZ235 in DHT-free medium (Fig. 5b).

## Discussion

Our previous study has demonstrated that ALDH1A3 is specifically expressed in luminal compartment in human prostate epitheliums. From the TCGA data of 333 primary prostate cancer, ALDH1A3 correlated with AR signaling pathway and corresponding luminal signature. It is also suggested that ALDH1A3 has a potential to be a predictor of survival in primary prostate cancer patients. In present study, with our single center follow up database, we performed IHC analysis on tissue microarray for patients with advanced disease upon adjuvant hormonal therapy after radical prostatectomy. The results showed that ALDH1A3 low-expression patients indicated shorter time to progression to castration resistance. The phenotype that we showed at the beginning has implications to understand the mechanisms of this prostate specific gene in the development of cancer progression. From the metastatic prostate cancer samples database, the RNA sequencing data demonstrated that ALDH1A3 was down regulated in mCRPC group compared with the primary prostate cancer. Next-generation sequencing (NGS) analysis has made it possible to reclassify different subtypes in a specific cancer by molecular changes. It indeed could provide benefits for clinical practice in oncology, such as diagnosis, prognosis, and treatment decisions. For example, patients with cancers of unknown primary (CUP), traditionally, are generally assumed to have a poor prognosis with a treatment in cytotoxic chemotherapy guided by histologic features and the pattern of metastatic spread. A new study showed that NGS may provide an opportunity for CUP patients to benefit from individualized therapies according to the targetable genomic alterations identified by tumor molecular profiling [16]. In that study, 10% of patients received targeted therapies based on their mutation signatures.

Thanks to the RNA sequencing data, we found that the PI3K pathway signature had been highly correlated with ALDH1A3 signature. Then we speculated the PI3K signaling pathway activation might be due to ADT resistance. And we also confirmed this hypothesis by showing the up-regulation of phospho-AKT upon ALDH1A3 knockout. Furthermore, PI3K signaling pathway inhibitor BEZ235 could rescue the ADT resistance following ALDH1A3 knockout. The PI3K/AKT/mTOR pathway is altered in almost 50% of mCRPC through either PTEN inactivation or/and aberrant activation in PIK3CA/B [13]. It’s been demonstrated that PI3K-AKT-mTOR signaling pathway deregulation resulting from PTEN loss is associated with androgen insensitivity and the development of CRPC [17]. Knocking down PTEN can convert the androgen-dependent Myc-CaP cell into androgen independence, suggesting that PTEN intrinsically controls androgen responsiveness, a critical step in the development of castration resistant prostate cancer [18]. Based on these data, phase I/II trials assessing the combination of next-generation AR therapy with a PI3K/AKT/mTOR inhibitor are currently ongoing (ClinicalTrials.gov identifier: NCT02407054 and NCT02215096). ALDH1A3, also as retinoic acid anabolizing enzyme [19] has been proved a potential novel target for triple-negative breast tumors and cancer stem cells [20]. Retinoic acid receptor-related orphan receptor γ (RORγ) antagonists are efficacious in re-sensitizing docetaxel and cabazitaxel cross-resistant CRPC cells [21]. It demonstrated that targeting retinoid signaling might be a potential approach in the treatment of CRPC [22].

## Conclusions

In conclusion, the NGS provides multiple new opportunities and tools to accelerate and facilitate the entire process of drug testing toward accelerated drug positioning and approval for precise and personalized medicine. It also allows researchers to discover some hidden pattern of the complex cancer. Such strategies include the development of inhibitors with a higher potency against their intended target, like ADT and Abiraterone, and the use of combination therapies incorporating inhibitors of parallel or alternative signaling pathways mediating acquired resistance, like the mTOR inhibitor BEZ235 in the treatment of PI3K-AKT-mTOR pathway activation. In this paper, we’ve investigated alterations in the targeted gene leading to ADT resistance to mCRPC. Based on the RNA sequencing and experimental results, we found that PI3K pathway alteration or activation might be the cause of the resistance. We, then, rescued the ADT resistance by PI3K pathway inhibitor-BEZ235. The acquired resistance to ADT therapy by some patients with low level of ALDH1A3 could be overcome by combination therapy with PI3K pathway inhibitor, which will provide a new potential approach to the treatment of mCRPC.

## Supplementary information

**Additional file 1 : Table S1:** A differential expression gene list as ALDH1A3 signature.

**Additional file 2 : Figure S1**: Original data of western blot (ALDH1A3) in Fig. 3a, the cropping of the blot by figure processing software was clearly mentioned with red rectangle.

**Additional file 3 : Figure S2**: Original data of western blot (GAPDH) in Fig. 3a, the cropping of the blot by figure processing software was clearly mentioned with red rectangle.

**Additional file 4 : Figure S3**: Original data of western blot (ALDH1A3) in Fig. 4, the cropping of the blot by figure processing software was clearly mentioned with red rectangle.

**Additional file 5 : Figure S4**: Original data of western blot (p-AKT) in Fig. 4, the cropping of the blot by figure processing software was clearly mentioned with red rectangle.

**Additional file 6 : Figure S5**: Original data of western blot (GAPDH) in Fig. 4, the cropping of the blot by figure processing software was clearly mentioned with red rectangle.

## Data Availability

The datasets analyzed in this study was included in supplementary Table 1.

## Abbreviations

mCRPC: metastatic Castration Resistance Prostate Cancer
GSEA: Gene set enrichment analysis
ADT: Androgen deprivation therapy
TCGA: The cancer genome atlas
SU2C: Stand up to cancer
NAD: Nicotinamide Adenine Dinucleotide
CSC: Cancer stem cell
TMAs: Tissue microarrays
LHRH: Luteinizing hormone-releasing hormone
RPKM: Reads Per Kilobase Million
DHT: Dihydrotestosterone
IHC: Immunohistochemistry
NGS: Next-generation sequencing
CUP: Cancers of unknown primary
RORγ: Retinoic acid receptor-related orphan receptor γ
