# Supplementary figures and images for: ALDH1A3 serves as a predictor for castration resistance in prostate cancer patients
Source: BMC Cancer. 2020 May 6;20:387. doi: 10.1186/s12885-020-06899-x (PMC7201787; doi:10.1186/s12885-020-06899-x)

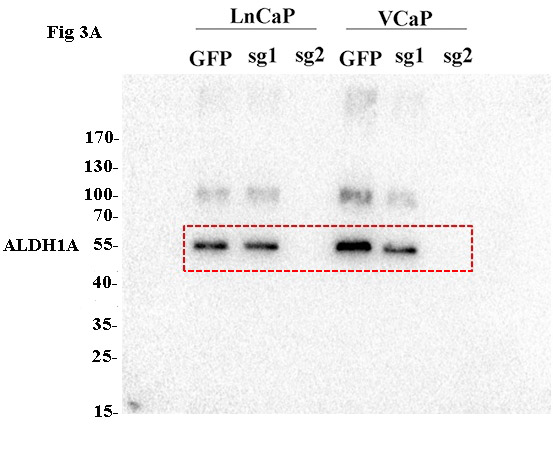

Supplement: Supplementary file 2 — Additional file 2 : Figure S1: Original data of western blot (ALDH1A3) in Fig. 3a, the cropping of the blot by figure processing software was clearly mentioned with red rectangle. [file 12885_2020_6899_MOESM2_ESM.jpg]

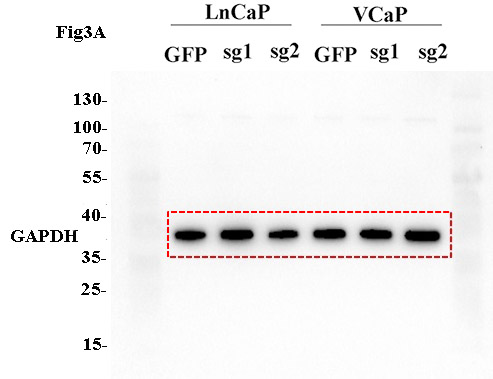

Supplement: Supplementary file 3 — Additional file 3 : Figure S2: Original data of western blot (GAPDH) in Fig. 3a, the cropping of the blot by figure processing software was clearly mentioned with red rectangle. [file 12885_2020_6899_MOESM3_ESM.jpg]

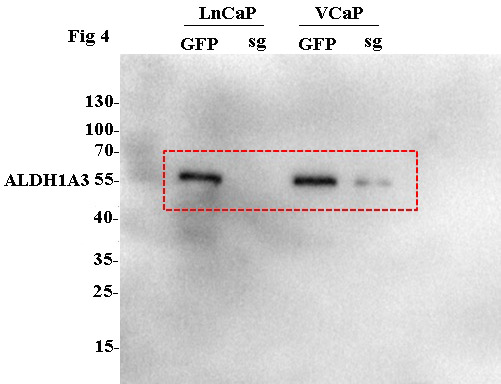

Supplement: Supplementary file 4 — Additional file 4 : Figure S3: Original data of western blot (ALDH1A3) in Fig. 4, the cropping of the blot by figure processing software was clearly mentioned with red rectangle. [file 12885_2020_6899_MOESM4_ESM.jpg]

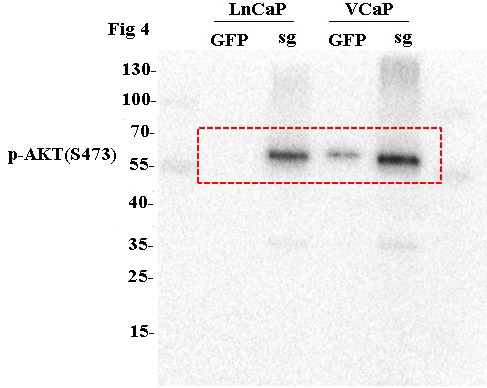

Supplement: Supplementary file 5 — Additional file 5 : Figure S4: Original data of western blot (p-AKT) in Fig. 4, the cropping of the blot by figure processing software was clearly mentioned with red rectangle. [file 12885_2020_6899_MOESM5_ESM.jpg]

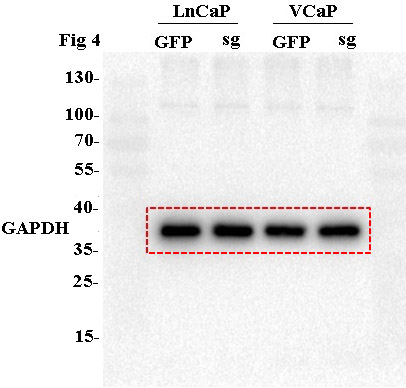

Supplement: Supplementary file 6 — Additional file 6 : Figure S5: Original data of western blot (GAPDH) in Fig. 4, the cropping of the blot by figure processing software was clearly mentioned with red rectangle. [file 12885_2020_6899_MOESM6_ESM.jpg]
